# A Preliminary Study Exploring the Relationship between Occupational Health Hazards and Gut Microbiota among Firefighters

**DOI:** 10.3390/life13091928

**Published:** 2023-09-18

**Authors:** Ji Youn Yoo, Daniel McSkimming, Kalavathy Rajan, Anujit Sarkar, Nicole Labbé, Maureen Groer, Usha Menon

**Affiliations:** 1 College of Nursing, University of Tennessee Knoxville, Knoxville, TN 37996, USA; asarkar7@utk.edu (A.S.); mgroer@utk.edu (M.G.); 2 Interdisciplinary Unit in Data Science & Analytics, Buffalo State University, Buffalo, NY 14222, USA; mcskimdi@buffalostate.edu; 3 Department of Plant and Soil Science, Fiber and Biopolymer Research Institute, Texas Tech University, Lubbock, TX 79403, USA; krajan@ttu.edu; 4 Center for Renewable Carbon, The University of Tennessee Institute of Agriculture, Knoxville, TN 37996, USA; nlabbe@utk.edu; 5 College of Nursing, University of South Florida, Tampa, FL 33612, USA; umenon@usf.edu

**Keywords:** chemical hazards, firefighters, gut microbiota, occupational hazards, post-traumatic stress disorder (PTSD), polycyclic aromatic hydrocarbons (PAHs)

## Abstract

Firefighters are exposed to occupational hazards and have a higher prevalence of health issues. The gut microbiota plays a crucial role in the immune, endocrine, and neural systems, and disruptions in its composition can impact health outcomes. This pilot study aimed to investigate the potential association between occupational factors, changes in gut microbiota, and the development of adverse health outcomes in firefighters. To test this hypothesis, we recruited 15 firefighters and age/sex-matched controls to investigate the relationship between occupational environment and gut microbiota. Firefighters exhibit lower intestinal bacterial alpha diversity and a higher presence of pathogenic bacteria than the control. Moreover, unique gut bacterial taxa were observed in firefighters with high post-traumatic stress disorder (PTSD) scores, which could contribute to immune dysregulation and higher susceptibility to pathogen colonization. These preliminary findings suggest that occupational factors, including exposure to traumatic stressors and chemicals, may influence firefighters’ health by modulating their gut microbiota. The observed changes in gut microbiota composition and the potential link to occupational hazards highlight the need for further research in larger sample-size studies. Understanding the role of gut microbiota in firefighter health may have implications for preventive measures and interventions to mitigate occupational health risks and improve overall well-being.

## 1. Introduction

Firefighters play a critical role in communities worldwide but are exposed to unique occupational hazards, including physical, chemical, and psychological stressors. Numerous studies have demonstrated that occupational factors, such as physical exertion and exposure to traumatic events, increase the risk of various health issues among firefighters, including post-traumatic stress disorder (PTSD), depression, anxiety, cardiovascular diseases, and cancer [1,2,3]. Exposure to traumatic stressors can have an impact on the brain–gut axis, a bidirectional communication system between the central nervous system and the gastrointestinal tract, including the gut microbiota [4]. Alterations in gut microbiota composition and diversity, known as gut dysbiosis, have been observed in populations with mental and physical health conditions. Although direct evidence specifically focusing on firefighters is limited, studies conducted on related professions or populations exposed to similar occupational hazards provide valuable insights. Research conducted on military personnel exposed to combat situations and high-stress environments has revealed associations between stress, gut dysbiosis, and various health outcomes [5,6]. Given the physically demanding nature of firefighting, exposure to combustion products, and the psychological stressors associated with traumatic events, it is plausible that these factors contribute to changes in gut microbiome among firefighters.

Moreover, studies have linked gut dysbiosis to specific occupational exposures, such as exposure to pollutants and chemicals [7]. Firefighters frequently encounter combustion byproducts containing potentially harmful substances like polycyclic aromatic hydrocarbons (PAHs) and other carcinogens [8]. Although research directly investigating the impact of these occupational exposures on firefighters’ gut microbiota is limited, studies on other occupational groups and animal studies exposed to similar chemicals have demonstrated associations between exposure, microbial dysbiosis, and adverse health effects [9].

Understanding the correlation between health risk factors in firefighters, changes in gut microbiota, and the development of adverse health outcomes is crucial. In this preliminary study, we aimed to investigate the relationship between firefighting occupational health risk factors, such as chemical accumulation levels and PTSD symptoms, and the structure of gut microbial communities. Specifically, we examined the microbial diversity between firefighters and non-firefighters, taking into account factors such as concentrations of PAHs (1-hydroxypyrene and benzo[a]pyrene), hair cortisol levels, and scores from various psychological self-questionnaires. By identifying significant differences in these factors between the two groups, we aimed to provide insights into the potential connection between occupational hazards, alterations in gut microbiota, and the increased risk of adverse health outcomes among firefighters.

## 2. Materials and Methods

### 2.1. Recruitment

**Figure 1:**
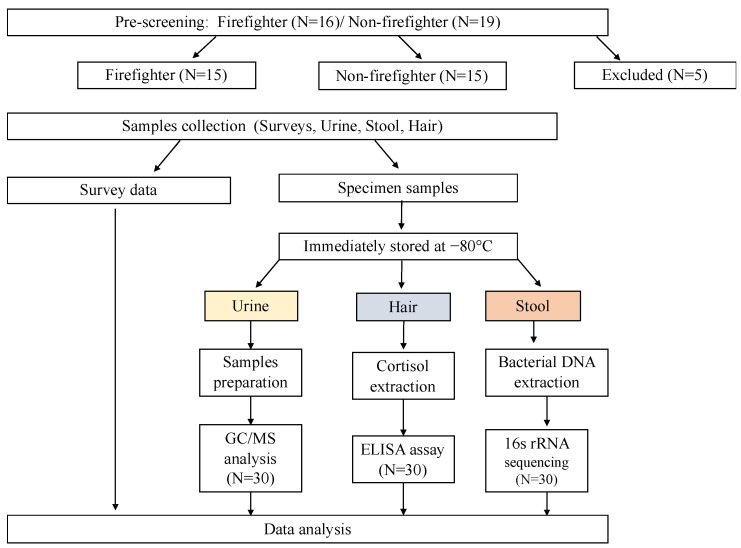
A flowchart of participants’ recruitment, sample collection, and processing. The study flyers were advertised on the university online portal, local churches and Tampa firefighter stations. A total of 35 individuals obtained an e-consent and answered the pre-screening survey. Only 30 individuals (firefighter: 15, non-firefighters: 15) met the study criteria. After obtaining informed consent, hair, urine, stool samples were collected and frozen immediately before being delivered to the University, College of Nursing Biobehavioral lab for further analysis.

Flyers and snowball sampling were used to recruit healthy male firefighters between 21 and 50 years of age, as well as a control group that was sex, geography, and age-matched, through fire stations and the university portal in Florida. A total of 35 participants were pre-screened. However, one firefighter with rheumatoid arthritis and four members of the control group with irritable bowel syndrome, inflammatory bowel disease, type 2 diabetes, or hepatitis C were excluded from the study. Therefore, the final analytical sample size consisted of 30 participants, with 15 healthy male firefighters and 15 controls (Figure 1). This study was approved by the University of South Florida Institutional Review Board (#Pro000410).

#### 2.1.1. Study Criteria

##### Inclusion Criteria

Firefighters: active firefighters with a minimum of 5 years of active service; aged 21–50 years; male; non-smokers, non-tobacco users, or those who stopped using tobacco 5+ years ago.

Controls: individuals who have never been a career or volunteer firefighter; meet the same demographic and health criteria as firefighters.

##### Exclusion Criteria

Firefighters: employed in 50% or higher administrative roles; diagnosed with chronic bowel disorders or inflammatory bowel disease; cancer history; diagnosed with human immunodeficiency virus, hepatitis A, hepatitis B, or hepatitis C; current user of pro/prebiotics; those who do not meet inclusion criteria.

Controls: meet the same health criteria as firefighters.

### 2.2. Sample and Data Collection

For pre-screening questionnaires, online consent was obtained in REDCap, where all study survey data were stored. Self-administered questionnaires, as well as stool, urine, and hair samples, were collected from each participant after written informed consent was obtained. Approximately 30 mg of hair was collected. Stool samples were collected using the Easy Stool Collection Kit (Alpco, Salem, NH, USA), and approximately 50 mL of mid-stream urine was collected into sterile urine containers (Norgen Bioteck, Thorold, ON, Canada). The participants were instructed to store all the samples immediately in a freezer until the collection day, which was less than 48 h after collection. A researcher visited fire stations or participants’ homes to collect the samples in an iced container to deliver to the lab.

### 2.3. Microbial DIVERSITY and COMPOSITION PROFILING by Illumina MiSeq

Stool bacterial DNA was extracted using the QIAamp DNA Stool Mini Kit (Qiagen, Germantown, MD, USA). We purified DNA for PCR amplification. The V4 region (primers 515F–806R) of the 16S rRNA gene was amplified and sequenced using the Illumina MiSeq platform to generate ~100,000 and 250 bp paired-end reads per sample. Using relevant R packages, the V4 region 16S rRNA gene amplicon reads were trimmed and processed using the DADA2 denoising pipeline. The taxonomy annotations were assigned against the SILVA v138 database. Least squares regression and non-parametric hypothesis tests were performed with both amplicon sequence variants (ASVs) and genus-level amalgamations to compare differences in alpha diversity (via Hill values), beta diversity (microbiome composition), and individual taxa across healthy firefighters and age, geography, and sex-matched controls. ASVs significant differences between the firefighters and the control samples were identified through MaAsLin2 (Microbiome multivariate association with linear models) [10].

### 2.4. Hair Cortisol Quantification by ELISA

Hair samples were weighed and cut into small pieces and ground for 25 s at 25 mH on the Retsch ball mill MM200 (Retsch, Haan, Germany). The powdered hair sample was removed, weighed, and placed in a glass scintillation vial. Methanol (2 mL) was added to the glass vials that contained hair powder, and the glass vials were incubated at room temperature for 24 h with slow rotation on a tabletop shaker. Following extraction, the solution was transferred to an Eppendorf tube and centrifuged for 10 min at 3000 rpm. The supernatant was removed, placed into drying tubes, and dried down in nitrogen for approximately 45 min at 37 °C while rotated at a speed of 30 rpm. The extracts were resuspended with 200 μL of ELISA assay buffer, and cortisol levels were determined using Salimetrics Salivary Cortisol ELISA kits (Salimetrics, State College, PA, USA) according to the manufacturer’s directions. Standards and controls were assayed along with the samples for each plate used to measure cortisol levels. All analyses were performed in duplicate.

### 2.5. Urine Analysis by Gas Chromatography-Mass Spectrometry (GC/MS)

Sample preparation for GC/MS was adapted from Campo et al., 2008 [11]. In the urine sample preparation process, 40 μL of internal standard (1 mg/mL benzo[a]pyrene-d12 solution from Fisher Scientific, Cleveland, OH, USA) was added to 6 mL of thawed urine samples. This mixture was vortexed with 3 g of magnesium sulfate, followed by liquid-liquid phase separation using 6 mL of n-hexanes twice. The resulting 12 mL of organic layer was combined in an amber vial and dried in a vacuum oven at 40 °C under reduced pressure. Subsequently, 100 μL of N, O-Bis(trimethylsilyl) trifluoroacetamide (from Sigma Aldrich, Saint Louis, MO, USA) was added to the amber vials, which were incubated at 45 °C for 90 min to derivatize the isolated PAH. After incubation, the samples were transferred to 2 mL amber autosampler vials with 400 μL glass inserts for analysis. The analysis was performed using an Agilent 7890 B GC system (Oak Ridge North, TX, USA) coupled with an Agilent 5977B single quadrupole detector (MSD). The GC/MS system utilized helium gas as the carrier at a flow rate of 1 mL/min. A total of 0.2 mL of the derivatized samples were injected, with the injection port temperature set at 300 °C. The column oven temperature started at 60 °C for 3 min, then ramped up to 150 °C, 210 °C, and 320 °C at a rate of 10 °C/min. The column was flushed with 15 mL/min of helium for 1 min at 320 °C between each sample. Mass fragmentation of compounds was recorded using electron ionization mode, with the ion source temperature set at 250 °C and the quadrupole mass filter temperature at 200 °C. Spectra were acquired within a scan range of 50–500 ion (*m/z*) value after a 6.8-min initial solvent delay. Peak integration and quantification were performed using Agilent’s MassHunter software. Benzo[a]pyrene and the internal standard peaks on the total ion chromatogram (TIC) were integrated, and benzo[a]pyrene was quantified by calculating the ratio of the detected peak size to the internal standard peak size against the known concentration of the internal standard (6.7 μg/mL) to account for potential extraction losses.

For multivariate analysis of the GC/MS spectra, principal component analysis (PCA) was conducted using Unscrambler X v10.4 software (Aspen Technology, Inc., Bedford, MA, USA). The total ion chromatograms of all control and firefighter urine samples (60 samples in total) were normalized based on the retention time of the internal standard peaks (35.46 min for benzo[a]pyrene-d12). The chromatograms were then further normalized according to the mean peak height and subjected to PCA. The PCA scores plot was used to group samples with similar GC/MS spectra, while the PCA loadings plot identified the times (*m/z* peaks) responsible for the grouping.

### 2.6. Self-Questionnaire Data Analysis

The demographic and psychological health characteristics of the participants, including the generalized anxiety disorder-2 (GAD-2), patient health questionnaire-2 (PHQ-2 Depression), perceived stress scale (PSS), and PTSD checklist—civilian version (PCL-C), were scored to indicate total scores for each variable. Each score was analyzed and compared using frequencies and descriptive statistics (IBM SPSS; Version 28.0 Armonk, NY, USA: IBM Corp.). The GAD-2, PHQ-2 Depression, PSS, and PTSD checklist (PCL-C) were completed by self-report questionnaire. When the participant had a score greater than or equal to 3 on either the GAD-2 or the PHQ-2, a referral for counseling was indicated. Participants with PSS scores ranging from 27 to 40 and PCL-C scoring over 45 were also provided a referral to a clinic. With only 15 participants per group, the statistical power was limited. Nevertheless, bivariate tests between gut microbial diversity, chemical exposures, and demographic and mental health characteristics were run. For all association tests, the significant association threshold was set at *p* = 0.05.

## 3. Results

### 3.1. Demographic Characteristics

The study recruited 30 healthy male participants aged 21 to 50, including firefighters and non-firefighter controls in South Florida. Age matching was performed with a plus or minus four-year age gap. Among firefighters, 73.3% were married compared to 60% in the control group. The majority of firefighters (83.3%) had a high school or college degree, while most individuals in the control group held a professional degree (60%). In terms of ethnicity, 86.7% of firefighters identified as White American, whereas in the control group, the percentage was 46.7%. Firefighters held various roles, including firefighter operators (27%), Captain or Chief Officer (40%), Driver engineer (20%), and Lieutenant (13%). In contrast, 60% of controls worked in the education/science field. While the exact number of fires each firefighter was exposed to is unknown, firefighters with over five years of experience were likely to have more fire exposure than non-firefighters. All firefighters had previously responded to suicide attempts and actual suicides, while the control group had only one such exposure. Regarding alcohol consumption, 14 firefighters (93%) reported consuming alcoholic beverages, compared to 11 non-firefighters (73%). The eleven firefighters had a higher-than-average intake of two alcoholic drinks per day, according to The National Institute on Alcohol Abuse and Alcoholism (NIAAA), whereas the non-firefighters had a 60% prevalence. In general, firefighters consumed more alcoholic beverages than the control group (Table 1).

### 3.2. Mental Health Self-Questionnaire and Hair Cortisol Data Analysis

**Figure 2:**
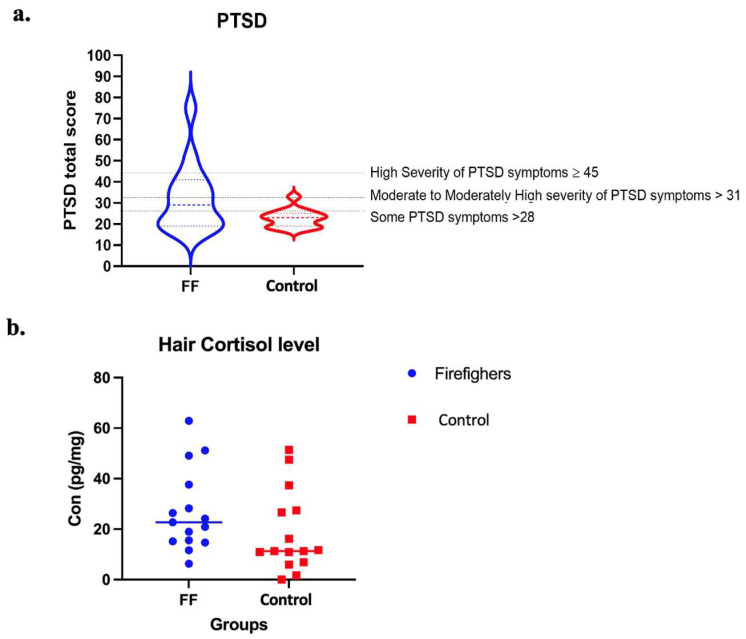
PTSD and hair cortisol levels. (**a**) The firefighter group shows a significantly higher total PTSD level (*p* = 0.003). (**b**) This graph shows hair cortisol levels in firefighters and control groups.

The mental health characteristics of the participants were analyzed using independent *t*-tests to compare the two groups. We added up all the PCL-C scores for each of the 17 items to calculate the total severity score, which ranged from 17 to 85. The total score was then categorized into four groups: (1) below 28, little to no severity; (2) 28–29, some PTSD symptoms; (3) 30–44, moderate to moderately high severity of PTSD symptoms; and (4) 45–85, high severity of PTSD symptoms. PCL-C total scores and individual questions were significantly higher in the firefighter group (*p* = 0.003), and 16 PCL-C questions were significantly high in firefighters Figure 2a. In contrast, the GAD-2 total score (*p* = 0.087) and PHQ-2 total score (*p* = 0.075) did not show significant differences between the groups. On the PSS scale, the total score (*p* = 0.04), Confident handling personal problems question (*p* = 0.006), and Piling up difficulties that one could not overcome questions (*p* = 0.02) were higher in firefighters compared to the control group.

Hair cortisol in firefighters (mean = 27) was slightly higher than in the control group (mean = 23.1), but it was not statistically significant (*p* = 0.3) (Figure 2b), and the coefficient of variation (CV) was 5%. We also ran bivariate tests between hair cortisol and health characteristics. We did not find any significant correlations between hair cortisol and mental health characteristics.

### 3.3. Microbial Diversity and Composition Profiling

**Figure 3:**
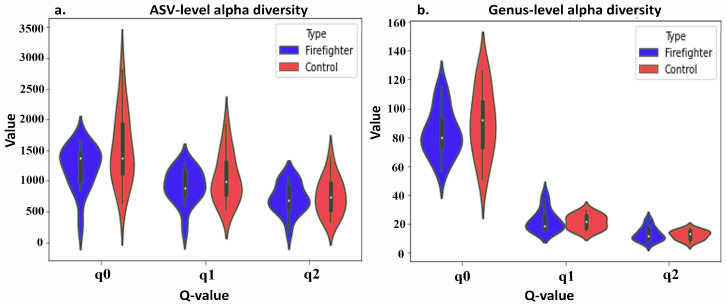
Alpha diversity (Hill values/number) between firefighter and control groups. Hill values (q = 0, species richness-based; q = 1, Shannon entropy-based; q = 2, Inverse Simpsons-based). (**a**) An amplicon sequence variant (ASV)-level alpha diversity. (**b**) Genus-level alpha diversity.

Alpha diversity was assessed using Hill values, a family of functions that offer advantages over standard diversity indices. Hill values represent the diversity of a sample as a measure of the effective number of equally abundant species that would yield the same diversity, providing a unit that allows for meaningful comparison between studies. We calculated the first three Hill values (q = 0, species richness-based; q = 1, Shannon entropy-based; q = 2, Inverse Simpsons-based) (Figure 3). Beta diversity was assessed using the *Bray–Curtis* distance (Supplementary Figure S1). We conducted a PERMANOVA analysis to examine the statistical significance of differences between the microbiota of the firefighter groups and the control group. We found no statistical significance within these two groups (*p* = 0.536), possibly due to the small sample size. However, we observed trends in the different relative abundances of alpha diversity at the ASV level (Figure 3), as well as in the composition of genera and phyla levels, including the phyla levels of *Actinobacteria* and genera levels of *Bifidobacterium*, which were more predominant in the control group than in the firefighters (Supplementary Figures S2 and S3).

**Figure 4:**
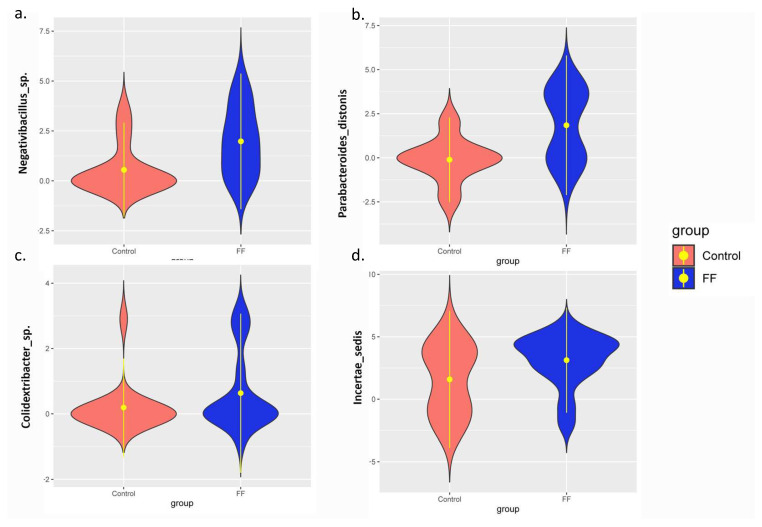

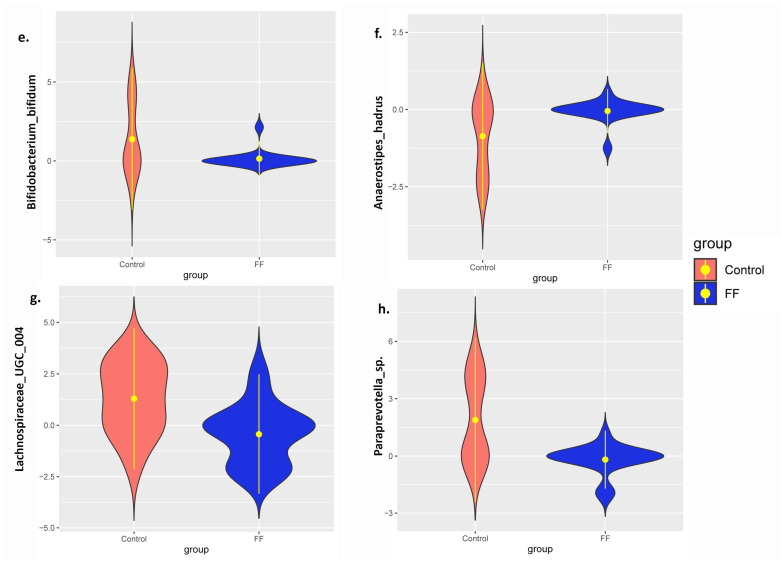
The amplicon sequence variants (ASVs represent bacterial taxons up to the genus/species level) whose abundances were significantly different between the firefighters and the control samples. The x-axis indicates the group, and the y-axis shows the CLR-corrected relative abundances of the important ASVs. (**a**) The abundance of *Negativibacillus* was significantly higher in the firefighters compared to the control group. (**b**) The abundance of *Parabacteroides distasonis* was significantly higher in the firefighters compared to the control group. (**c**) The abundance of *Colidextribacter* was significantly higher in the firefighters compared to the control group. (**d**) The abundance of *Incertae sedis* was significantly higher in the firefighters compared to the control group. (**e**) The abundance of *Bifidobacterium bifidum* was significantly lower in the firefighters compared to the control group. (**f**) The abundance of *Anaerostipes hadrus* was significantly lower in the firefighters compared to the control group. (**g**) The abundance of *Lachnospiraceae UCG-004* was significantly lower in the firefighters compared to the control group. (**h**) The abundance of *Paraprevotella* was significantly lower in the firefighters compared to the control group.

In the next step, we examined the association of ASVs to groups (firefighters vs. controls) employing MaAsLin2, which applies a generalized regression model and has been found to be efficient in handling the microbiome data. The abundance of *Negativibacillus* (*p* = 0.004), *Parabacteroides distasonis* (*p* = 0.001), *Colidextribacter* (*p* = 0.034), and *Incertae sedis* (*p* = 0.035) was significantly higher in the firefighters compared to the control group. The abundance of *Bifidobacterium bifidum* (*p* = 0.019), *Anaerostipes hadrus* (*p* = 0.028), *Lachnospiraceae UCG-004* (*p* = 0.013), and *Paraprevotella* (*p* = 0.009) was significantly lower in the firefighters compared to the control group (Figure 4). Table 2 displays some of the ASVs that were identified among a total of 83, with a *p*-value of ≤0.05.

**Figure 5:**
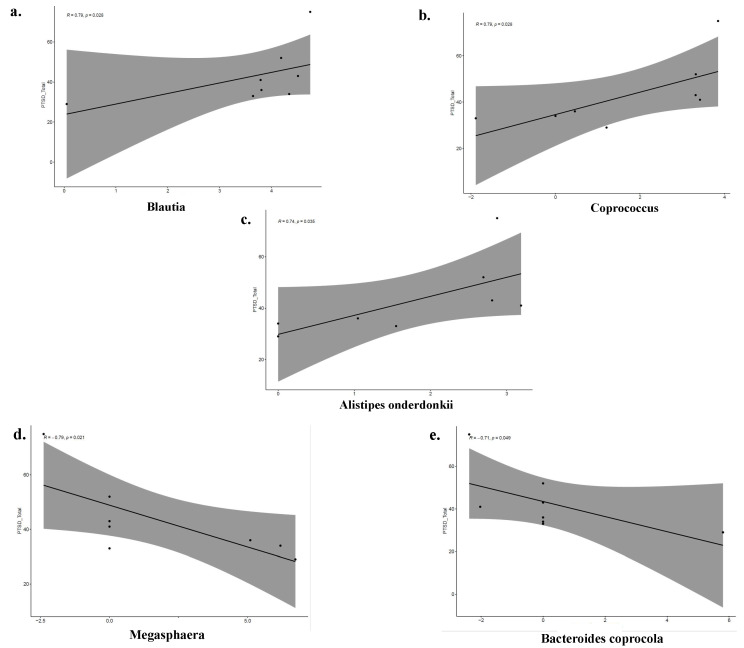
The correlation between *Lachnospiraceae blautia* and total PCL-C scores. (**a**) The correlation between *Blautia* and total PCL-C scores. (**b**) The correlation between *Coprococcus* and total PCL-C scores. (**c**) The correlation between *Alistipes onderdonkii* and total PCL-C scores. (**d**) The correlation between *Megasphaera* and total PCL-C scores. (**e**) The correlation between *Bacteroides coprocola* and total PCL-C scores.

We observed an interaction between the abundance of certain bacteria taxa and a total PCL-C score above 28, indicating moderate to severe PTSD symptoms in firefighters. In each linear regression model, total PCL-C scores were the dependent variable, while the independent variable was taxa abundance (Figure 5). *Lachnospiraceae blautia* (R = 0.79 *p* = 0.028), *Lachnospiraceae coprococcus* (R = 0.79, *p* = 0.028), and *Alistipes onderdonkii* (R = 0.74, *p* = 0.035) were positively correlated with total PTSD symptom scores in the firefighter with the total PCL-C score above 28. Conversely, *Veillonellaceae megasphaera* (R = −0.79, *p* = 0.021) and *Bacteroides coprocola* (R = −0.71, *p* = 0.049) were negatively associated with total PTSD symptom scores.

### 3.4. Urinary PAH Determination and Comparison

Of the two targeted PAH compounds, i.e., 1-OHP and benzo[a]pyrene, only the latter was detected by GC/MS in the urine samples. Benzo[a]pyrene was detected in 7 out of the 15 firefighters but not in any of the non-firefighters (Table 3). When considering the subgroups by firefighter role, the highest benzo[a]pyrene concentration was detected in lieutenants (≥982 g/L), followed by operators (≥711 g/L), driver/engineers (≥392 g/L), and finally captains/chiefs (≥241 g/L). Specifically, the lieutenant position had a much higher mean benzo[a]pyrene concentration than captain/chief (*p* = 0.03). There was no significant difference (*p* = 0.17) between the means of benzo[a]pyrene concentration and the years of firefighting service, nor the firefighter’s age (*p* = 0.41). Further investigation using a larger sample size is warranted to determine whether this occupation-based trend is significant.

**Figure 6:**
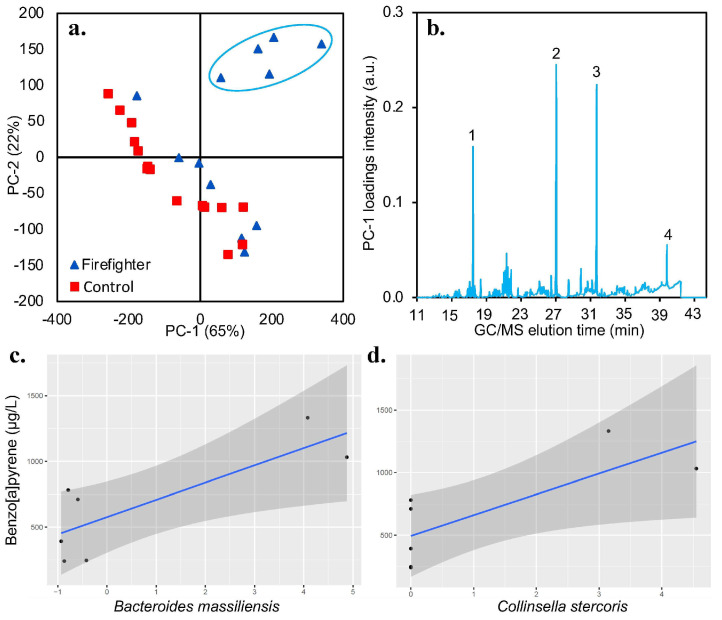
Principal component analysis (PCA) of urinary GC/MS spectra of firefighter and control groups. (**a**) PCA scores depicting the grouping of urine samples according to differences in chemical makeup; significantly different samples are highlighted using a manually drawn ellipse. (**b**) PCA loadings plot depicting the TIC (total ion chromatogram) peaks responsible for sample grouping along the principal component-1 (PC-1). Legend for TIC peaks: ① 3-(3,5-di-tert-butyl-4-hydroxyphenyl) propionate; ② 3-ethoxy-4-methoxyphenyl(6-methyl-1,3-benzodioxol-5-yl) ketone; ③ palmitic acid; ④ benzo[a]pyrene. (**c**) The correlation between benzo[a]pyrene concentration and *Bacteroides massiliensis*. (**d**) The correlation between benzo[a]pyrene concentration and *Collinsella stercoris*.

A multivariate technique like PCA was used to statistically determine the separation between firefighters and controls according to their urine chemical makeup. The urinary metabolites of control individuals were not significantly different from each other, as seen by their distribution in the PCA scores plot (Figure 6a). However, However, there was a high variation among firefighters. Specifically, the positive quadrant of the PCA scores plot (Figure 6a) shows that a small group of firefighters had 65% (PC-1 scores) different urine composition than other firefighters as well as the control group. The PC-1 loadings plot (Figure 6b) shows the TIC peaks that gave rise to the 65% variance in the subset of firefighters circled in Figure 6a; the four significantly intense TIC peaks corresponded to benzo[a]pyrene, 3-(3,5-Di-tert-butyl-4-hydroxyphenyl) propionate, 3-Ethoxy-4-methoxyphenyl(6-methyl-1,3-benzodioxol-5-yl) ketone, and palmitic acid. Other than benzo[a]pyrene, which is a common carcinogen in smoke and soot, a significant finding here is the exposure of this subset of firefighters to 3-(3,5-Di-tert-butyl-4-hydroxyphenyl) propionate, which is a derivative of butylated hydroxytoluene (BHT) plastics found in fireproofing plasticizers [12]. We also conducted a correlation analysis between the benzo[a]pyrene concentration quantified in the seven firefighters (Table 3) and their gut microbial taxa. Our results, as seen in Figure 6c,d, shows that the concertation of benzo[a]pyrene in urine has a positive association with tumor tissue-specific bacterial biomarker panels, *Bacteroides massiliensis* (R = 0.8, *p* = 0.027) and *Collinsella stercoris* (R = 0.77, *p* = 0.042), found in colorectal cancer patients [13].

## 4. Discussion

Firefighters face higher health risks, such as chemical exposure, psychological trauma, and a higher likelihood of developing certain types of cancer due to their occupation. PTSD is a condition that can result from exposure to traumatic events, evoking feelings of horror, fear, or helplessness. Symptoms include flashbacks, difficulty recalling details, avoidance of triggers, loss of interest, emotional detachment, heightened arousal, and increased irritability [14]. While affecting 1% to 8% of the general population, firefighters have a significantly higher rate, ranging from 17% to 22%, due to their exposure to hazardous workplace situations [15]. Our study also confirms that eight out of fifteen firefighters had moderate to severe PTSD symptoms (PCL-C above 28), whereas only one control had moderate PTSD symptoms. Furthermore, there is increasing evidence that firefighters are exposed to repeated and prolonged severe traumatic events during their duties, which could lead to a more considerable risk for PTSD [15]. The combination of repeated exposure to traumatic stressors, among other occupational factors, contributes to the development of PTSD [16]. For example, Donnelly et al. (2009) suggested that several demographic factors, including marital status, age, and educational status, are associated with PTSD symptoms [16]. Accordingly, our study shows a negative correlation between educational status and PTSD symptom scores and a positive correlation between alcohol consumption and PTSD symptoms scores.

Exposure to severe traumatic events could be linked with the subsequent development of physical and behavioral health problems as well as many other mental diseases [17,18]. Our findings also show the positive correlation between the total PTSD symptoms score and the score of depression, anxiety, and perceived stress levels. Given the limitations of our study, further research with larger sample sizes may be needed to confirm our findings and explore potential underlying mechanisms. Nonetheless, our study adds to the existing literature on the associations between PTSD symptoms and educational status, alcohol consumption, and comorbidity with other mental health problems. It underscores the importance of addressing traumatic events and their potential negative consequences on mental health.

Cortisol is a hormone that the body releases in response to stress. Hair cortisol levels can represent chronic stress exposure over an extended period, serving as a measure of hypothalamic–pituitary–adrenal (HPA) axis functioning. The HPA axis is one of the major systems involved in the stress response, releasing corticotropin-releasing hormone (CRH), adrenocorticotropic hormone (ACTH), and cortisol, bringing the body back to homeostasis. However, chronic or prolonged stress can interrupt this feedback system. [19]. Several studies have investigated the correlation between hair cortisol levels and PTSD or PTSD symptoms in human subject studies. Some studies indicate that individuals with a history of trauma have reduced hair cortisol levels, while others suggest that ongoing exposure to stress can result in higher hair cortisol levels [20,21]. This discrepancy could potentially be attributed to other confounding factors, including variables like age, the method of hair segmentation, and the timing of sample collection. Moreover, the absence of a standardized unit of measurement for reporting hair cortisol levels presents an additional challenge in interpreting the results [22]. Therefore, ensuring the measurement of hair cortisol levels while considering other confounding factors is crucial within a study to precisely assess HPA axis activity. Although we controlled for the most influential potential confounding factors, including age, sex, and the length of hair, to determine hair cortisol concentration, our study findings only indicate a trend of increasing hair cortisol concentration in firefighters. This is intriguing since the firefighter group has a higher PTSD score than the control group, leading us to anticipate statistical significance in hair cortisol concentration between the groups. However, the comparison did not yield statistically significant results when compared to the control group. This could be affected by the small sample size. Therefore, further investigations are needed.

The brain–gut axis is one of the most vital bidirectional complex communications between the gut and brain that responds to stressors [23]. Recent data indicate that individuals with PTSD and depressive disorder showed increased brain–gut axis dysfunctions and increased activation of pro-inflammatory cytokines [24]. Consequently, the brain–gut axis has been highlighted as a critical therapeutic target for mental disorders, including PTSD, depression, and anxiety. Individuals with PTSD have a higher risk for altered HPA axis function, which involves corticotropin-releasing hormone (CRH), one of the stress response mediators in the brain–gut axis [25]. CRH is released from the hypothalamus and stimulates the secretion of adrenocorticotropic hormone from the pituitary gland, which in turn regulates the gastrointestinal immune reaction and stimulates more proinflammatory cytokines [26]. For example, CRH-receptor 1 (CRHR1) causes intestinal injury by activating intestinal inflammation, increasing gut permeability, and changing gut microbiota and morphology [26]. In addition, PTSD is associated with increased proinflammatory cytokines levels, plasma C-reactive protein (CRP), and glucocorticoid (GC) sensitivity of immunologic tissues [27,28]. Although the brain–gut axis theory is still controversial, researchers have argued that stress could alter the gut microbiota and promote the growth of more pathogenic bacteria [29,30]. The gut microbiota is an essential mediator for the brain–gut axis and the host immune system. This complex network tells us that exposure to traumatic stressors plays an essential role in the relationship between the brain, the intestine, and inflammation.

Alterations of the gut microbiota have been observed with various disease conditions, allowing pathogens to enter the circulation and cause human illness by regulating the metabolic, endocrine, and immune systems [31]. We also found patterns of association between firefighters with higher PTSD symptom scores and changes in gut microbiota, significantly reduced SCFA-producing bacteria, and increases in known pathogenic bacterial taxa. For example, our firefighter group showed a lower abundance of *Lachnospiraceae UCG-004* compared to the control group. *Lachnospiraceae* are known to produce SCFAs [32], especially butyrate, which play a role in improving intestinal barrier function and enhancing mucosal immunity [33]. Toya et al. (2020) also reported that a lower abundance of *Lachnospiraceae UCG-004* was significantly associated with 53 advanced coronary artery disease patients compared to the control group [34]. *Parabacteroides distasonis*, an anaerobic bacterium in the gastrointestinal tract, was predominant in our firefighters’ stool samples compared to the control. *Parabacteroides distasonis* has been shown to have a potential pathogenic role in multi-drug resistance and Crohn’s disease [35]. Yet, other contradictory findings suggest that *Parabacteroides distasonis* could be beneficial and have anti-disease functions against type 2 diabetes, sclerosis, and inflammatory bowel disease [36].

We also found a correlation between total PTSD symptoms score (above 28) and specific gut microbial taxa, including *Lachnospiraceae blautia*, *Lachnospiraceae coprococcus*, *Rikenellaceae alistipes onderdonkii*, *Veillonellaceae megasphaera,* and *Bacteroides coprocola*, in firefighters but not in the control group. *L. Blautia*, recently discovered as a genus within *Lachnospiraceae*, predominantly occurs in mammals’ intestines and feces. It is recognized as one of the bacteria with the potential for probiotic effects, such as fermenting dietary fiber, producing SCFAs, and influencing energy metabolism [37]. One study indicates a correlation between depression symptom scores and *L. Blautia* ASVs. This study demonstrated that even ASVs from the same genus could show opposite associations with depression symptom scores, displaying both significant positive and significant negative correlations with levels of depressive symptoms [38]. This discrepancy might arise when we only possess information about the genus level of a bacteria, as different species within the same genus might have distinct functions. Therefore, it is important to examine bacterial functions at the species level in psychological health conditions, including PTSD.

Another microbiome study using the 16S rRNA sequencing approach showed that *Lachnospiraceae* at the genus level, specifically *L. coprococcus*, and *Rikenellaceae*, both known as butyrate producers, were less abundant in individuals with major depressive disorder [39]. However, our study indicated the opposite trend. It could be possible to observe these differences at the family or genus level of bacterial taxa. Therefore, further studies should identify the specific gut microbiota species that disproportionately contribute to the psychological health of firefighters. *Veillonellaceae* has been associated with exacerbating inflammation (IL-13, IL-6) and higher prevalence in patients with hepatic encephalopathy [40] and irritable bowel syndrome patients [41]. An increased relative abundance of *Veillonellaceae* was observed in individuals with schizophrenia and was negatively associated with the severity of the disease [42]. Our results also revealed a negative correlation between PTSD symptoms and *Megasphaera*, a genus within the *Veillonellaceae* family. Another study on schizophrenia found that the relative abundances of the *Veillonellaceae megasphaera* were increased in individuals with chronic antipsychotic treatment compared to those with first-episode drug-naïve schizophrenia [43]. However, it is important to note that limited research has been conducted regarding PTSD and the gut microbiome.

While no specific causative relationship has been established between these bacteria and PTSD or any other mental health conditions, studies, including our own, have investigated alterations in gut microbiota composition in individuals with psychological disorders. Such changes to the microbiome provide a cornerstone for understanding how the occupational environment of firefighters may affect gut microbial diversity. Further research should uncover the complex network and linked mechanisms between exposure to psychological stress and negative health outcomes.

Benzo[a]pyrene, one of the most well-known occupationally relevant carcinogens, was only detected in high concentrations in firefighters, and it was positively associated with *Bacteroides massiliensis*. Hasan et al. (2022) suggest that *Bacteroides massiliensis* can be used as a potential biomarker for colorectal cancer diagnosis and prognosis, while other studies reported a higher relative abundance of *Bacteroides massiliensis* in prostate cancer patients [35]. As such, the gut dysbiosis observed in firefighters may be the result of a combination of multiple factors. Additionally, the urinary chemical compounds revealed by multivariate analysis to be differentially observed in firefighters might be targets for further research into these microbiota-wide alterations. While these compounds are poorly characterized in past research, their presence might contribute to altered health outcomes as well.

## 5. Conclusions

Our study provides a broad preliminary picture of the gut microbiota interactions with PTSD symptom scores. Exposure to occupational hazards such as traumatic events and carcinogens leads to the risk of gut dysbiosis and, in turn, to any associated adverse health outcomes. Thus, our findings support the beginning hypothesis that the disproportionate risk for occupation-related morbidities in firefighters is linked to gut microbiota changes. We will use these findings to inform future research. While the small sample size and other limitations to data collection (e.g., diet) preclude definitive results, we did demonstrate the feasibility of recruiting firefighters and obtaining physical samples. To further investigate our central hypothesis, we are recruiting a larger sample size.

## 6. Limitations

A limitation of this manuscript is the small sample size used in the study. Additionally, the study did not collect data on other potential influential factors that could affect the gut microbiome. Further investigation is required with a larger sample size.

## Figures and Tables

**Table 1:** Demographic characteristics of both firefighter and control groups.

Characteristics	Firefighters	Non-Firefighters	*p*-Value
**Age (Years)**	**Number (%) ** ^†^		
Age ranges from 21 to 30	3 (20%)	4 (27%)	1
Age ranges from 31 to 40	8 (53%)	5 (33%)	0.46
Age ranges from 41 to 50	4 (27%)	6 (40%)	0.7
**Marital Status**			
Married	11 (73%)	9 (60%)	0.7
Divorced or separated	1 (7%)	-	1
Widow	1 (7%)	-	1
Never Married	2 (13%)	6 (40%)	0.21
**Education**			
High school diploma	5 (33%)	1 (7%)	0.17
College degree (associate’s, bachelor’s)	9 (60%)	5 (33%)	0.27
Professional degree (Master’s, PhD)	1 (7%)	9 (60%)	0.005
**Ethnicity/Race ** *			
White	13 (87%)	7 (47%)	0.005
Asian American	-	6 (40%)	0.017
Black/African American	2 (13%)	2 (13%)	1
Occupational positions	4 (27%)	-	0.1
Firefighter operators	6 (40%)	-	0.017
Captain or Chief Officer	3 (20%)	-	0.22
Driver engineer	2 (13%)	-	0.48
Lieutenant	-	9 (60%)	0.02
Education/Science	-	4 (27%)	0.1
Industry Religion		2 (13%)	0.48
**Experience of suicide attempt or completion experiences situation**	15 (100%)	1 (7%)	2 × 10^−7^
**Smoking**			
Never smoked any tobacco	10 (67%)	10 (67%)	1
Quitted more than five years ago	5 (33%)	3 (20%)	0.68
Quit smoking in the last five years	-	2 (13%)	0.48
**Consuming alcoholic beverages**			
Yes	14 (93%)	11 (73%)	0.33
No	1 (7%)	4 (27%)	
**How many alcoholic beverages are consumed**			
More than 2 alcoholic drinks per day	11 (73.4%)	9 (60%)	0.7

^†^ Values in parentheses represent the percentage out of *N* = 30. The sample size included 15 firefighters and 15 non-firefighters, and there were no missing values. * Other race/ethnic groups were not included.

**Table 2:** Association between ASVs and the population groups (firefighters vs. controls).

Bacterial Taxa	Coefficient	*p*-Value
*k_ Bacteria; p_ Firmicutes; c_ Clostridia; o_ Oscillospirales; f_ Ruminococcaceae; g_ Negativibacillus; s_*	0.003	0.004
*k_ Bacteria; p_ Bacteroidota; c_ Bacteroidia; o_ Bacteroidales; f_ Tannerellaceae; g_ Parabacteroides; s_ distasonis*	0.031	0.001
*k_ Bacteria; p_ Firmicutes; c_ Clostridia; o_ Oscillospirales; f_ Oscillospiraceae; g_ Colidextribacter; s_*	0.01	0.034
*k_ Bacteria; p_ Firmicutes; c_Clostridia; o_Oscillospirales; f_Ruminococcaceae; g_Incertae_Sedis; s_*	0.027	0.035
*k_Bacteria; p_Bacteroidota; c_Bacteroidia; o_Bacteroidales; f_Bacteroidaceae; g_Bacteroides; s_cellulosilyticus*	0.013	0.01
*k_Bacteria; p_Firmicutes; c_Clostridia; o_Lachnospirales; f_Lachnospiraceae; g_[Eubacterium]_hallii_group; s_*	0.023	0.015
*k_Bacteria; p_Firmicutes; c_Clostridia; o_Oscillospirales; f_Ruminococcaceae; g_Faecalibacterium; s_*	−0.003	0.012
*k_Bacteria; p_Actinobacteriota; c_Actinobacteria; o_Bifidobacteriales; f_Bifidobacteriaceae; g_Bifidobacterium; s_bifidum*	−0.019	0.019
*k_Bacteria; p_Firmicutes; c_Clostridia; o_Lachnospirales; f_Lachnospiraceae; g_Anaerostipes; s_hadrus*	−0.001	0.028
*k_ Bacteria; p_ Firmicutes; c_ Clostridia; o_ Lachnospirales; f_ Lachnospiraceae; g_ Lachnospiraceae_UCG-004; s_*	−0.011	0.013
*k_Bacteria; p_Bacteroidota; c_Bacteroidia;Bacteroidales; o_Prevotellaceae; f_Paraprevotella; g_; s_*	−0.025	0.009
*k_Bacteria; p_Firmicutes; c_Clostridia; o_Lachnospirales; f_Lachnospiraceae; g_Blautia; s_*	−0.005	0.006

Each ASV represents a bacterial taxon up to the genus/species level, whose full classification is shown in the first column. The coefficient column indicates a measure of the size of the fold change between the firefighters and control groups. The corresponding *p*-values suggest that the difference was statistically significant (*p* ≤ 0.05). All results were obtained from the output of MaAsLin2. Only a few of the top significant associated ASVs are shown here. The taxonomy of all ASVs is shared at https://doi.org/10.6084/m9.figshare.22199464 (accessed on 13 September 2023).

**Table 3:** Benzo[a]pyrene concentration in the urine samples of firefighters and control individuals determined using GC/MS. * Firefighter urine sample represents sample ID from FF2 to FF16, and the control group samples were given ID from FF20 to FF37. The detection limit was 50 μg/L for Benzo[a]pyrene using GC/MS. “n.d.” indicates “not detectable”. Benzo[a]pyrene was detected only in firefighter urine samples such as sample ID FF 2, 6, 7, 8, 9, 10, and 12. The compound was not detected in any of the non-firefighter controls.

	Sample ID *	Concentration μg/L	Number	Sample ID	Concentration μg/L	Number	Sample ID	Concentration μg/L
1	FF2	241.2	11	FF12	781.7	21	FF26	n.d.
2	FF3	n.d.	12	FF13	n.d.	22	FF28	n.d.
3	FF4	n.d.	13	FF14	n.d.	23	FF30	n.d.
4	FF5	246.6	14	FF15	n.d.	24	FF31	n.d.
5	FF6	1332.3	15	FF16	n.d.	25	FF32	n.d.
6	FF7	1031.0	16	FF20	n.d.	26	FF33	n.d.
7	FF8	392.6	17	FF21	n.d.	27	FF34	n.d.
8	FF9	711.4	18	FF22	n.d.	28	FF35	n.d.
9	FF10	982.3	19	FF24	n.d.	29	FF36	n.d.
10	FF11	n.d.	20	FF25	n.d.	30	FF37	n.d.

## Data Availability

Taxonomy of all ASVs observed: https://doi.org/10.6084/m9.figshare.22199464 (accessed on 13 September 2023). Metadata of all samples: https://doi.org/10.6084/m9.figshare.22199458 (accessed on 13 September 2023). ASV counts for all samples: https://doi.org/10.6084/m9.figshare.22199431 (accessed on 13 September 2023).

## Supplementary Materials

The following supporting information can be downloaded at: https://www.mdpi.com/article/10.3390/life13091928/s1, Figure S1. Beta diversity: non-metric multidimensional scaling (NMDS) plot for firefighter and control samples. Figure S2. Relative abundances at the phyla level: stacked barplot representing the phyla distribution in all the samples. Figure S3. Relative abundances of the top 15 genera: stacked barplot representing the genera distribution in all the samples.

## Abbreviations

ASVs	Amplicon Sequence Variants
CLR	Center Log-Ratio
CRC	Colorectal Cancer
CRH	Corticotropin-Releasing Hormone
GAD-2	Generalized Anxiety Disorder-2
HPA axis	Hypothalamic–Pituitary–Adrenal axis
IBS	Irritable Bowel Syndrome
IBD	Inflammatory Bowel Disease
PHQ-2	Patient Health Questionnaire-2
PSS	Perceived Stress Scale
PAH	Polycyclic Aromatic Hydrocarbon
PTSD	Post-Traumatic Stress Disorder
PCA	Principal Component Analysis
PCL-C	PTSD CheckList–Civilian Version
TIC	Total Ion Chromatogram
