# Supplementary material for: A Preliminary Study Exploring the Relationship between Occupational Health Hazards and Gut Microbiota among Firefighters
Source: Life (Basel). 2023 Sep 18;13(9):1928. doi: 10.3390/life13091928 (PMC10533145; doi:10.3390/life13091928)

Supplementary Figure S1. Beta diversity: Non-metric Multidimensional Scaling (NMDS) plot for firefighter and control samples.

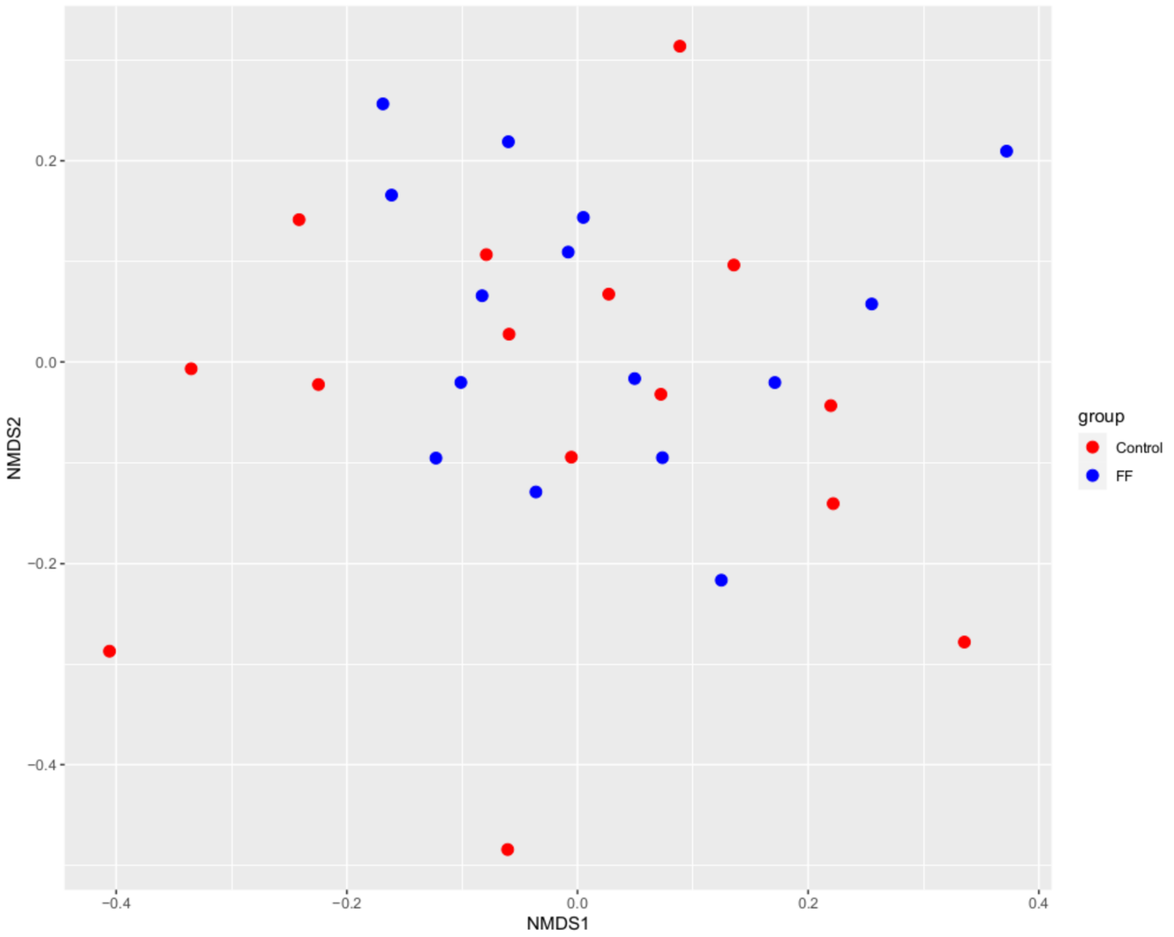

Supplementary Figure S2. Relative abundances at the phyla level

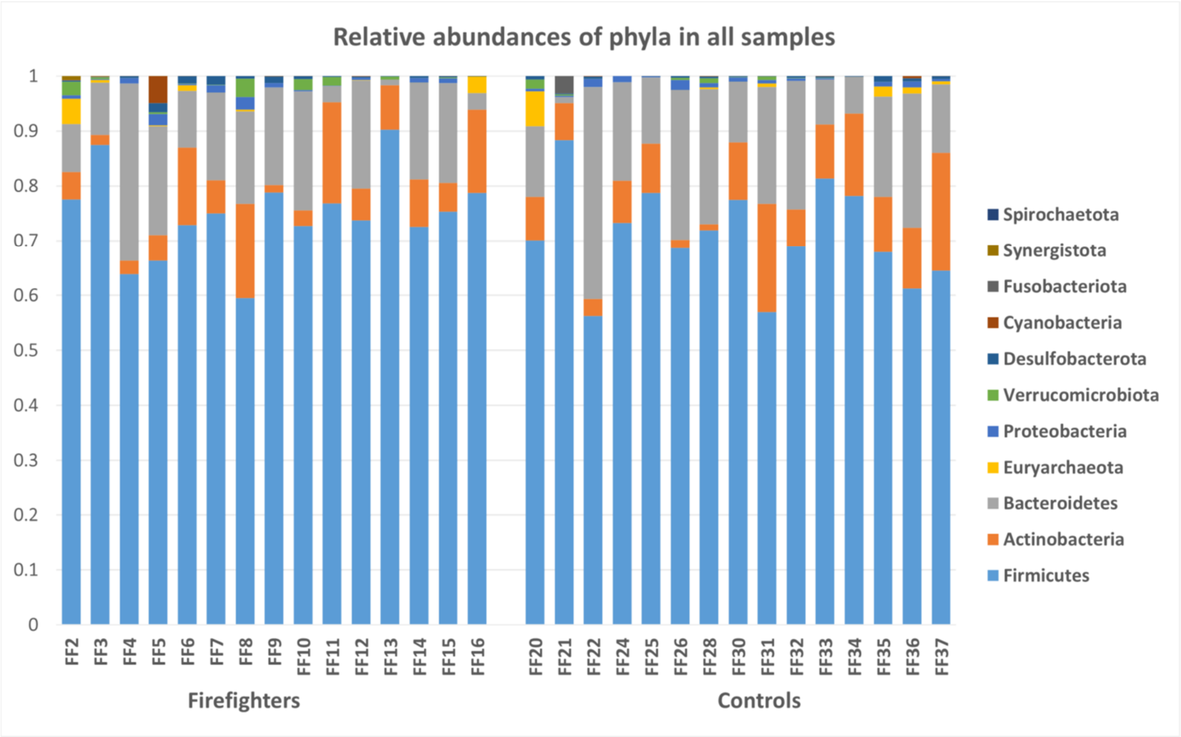

Supplementary Figure S3. Relative abundances of the top 15 genera

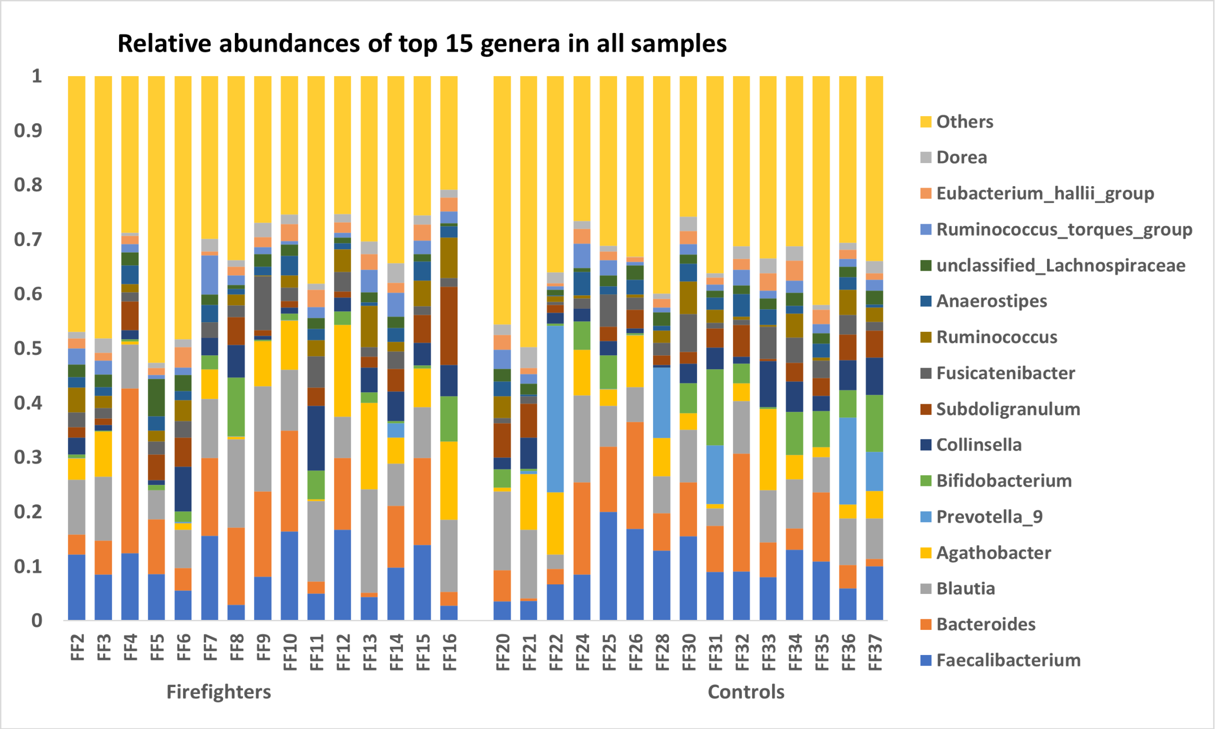

Supplement: Supplementary file 1 [file life-13-01928-s001.zip › life-2589163-supplementary.pdf]
